# Prevalence of Cam Morphology in Females with Femoroacetabular Impingement

**DOI:** 10.3389/fsurg.2015.00061

**Published:** 2015-12-01

**Authors:** David M. Levy, Michael D. Hellman, Joshua D. Harris, Bryan Haughom, Rachel M. Frank, Shane J. Nho

**Affiliations:** ^1^Department of Orthopedic Surgery, Hip Preservation Center, Rush University Medical Center, Chicago, IL, USA; ^2^Houston Methodist Hip Preservation Center, Houston Methodist Orthopedics & Sports Medicine, Houston Methodist Hospital, Houston, TX, USA

**Keywords:** femoroacetabular impingement, cam, female, alpha angle, head–neck offset

## Abstract

Cam and pincer are two common morphologies responsible for femoroacetabular impingement (FAI). Previous literature has reported that cam deformity is predominantly a male morphology, while being significantly less common in females. Cam morphology is commonly assessed with the alpha angle, measured on radiographs. The purpose of this study is to determine the prevalence of cam morphology utilizing the alpha angle in female subjects diagnosed with symptomatic FAI. All females presenting to the senior author’s clinic diagnosed with symptomatic FAI between December 2006 and January 2013 were retrospectively reviewed. Alpha (α) angles were measured on anteroposterior and lateral (Dunn 90°, cross-table lateral, and/or frog-leg lateral) plain radiographs by two blinded physicians, and the largest measured angle was used. Using Gosvig et al.’s classification, alpha angle was characterized as (pathologic > 57°), borderline (51–56°), subtle (46–50°), very subtle (43–45°), or normal (≤42°). Three hundred and ninety-one patients (438 hips) were analyzed (age 36.2 ± 12.3 years). Among the hips included, 35.6% were normal, 14.6% pathologic, 15.1% borderline, 14.6% subtle, and 20.1% very subtle. There was no correlation between alpha angle and patient age (*R* = 0.17) or body mass index (*R* = 0.05). The intraclass correlation coefficient for α-angle measurements was 0.84. Sixty-four percent of females in this cohort had an alpha angle >42°. Subtle cam deformity plays a significant role in the pathoanatomy of female patients with symptomatic FAI. As the majority of revision hip arthroscopies are performed due to incomplete cam correction, hip arthroscopists need to be cognizant of and potentially surgically address these subtle lesions.

## Introduction

Femoroacetabular impingement (FAI) is a pathologic condition described by Ganz et al. (1) in which there is abnormal contact between the femoral head and acetabulum leading to hip pain, labral tears, chondral injuries, and early osteoarthritis (1–8). The two most common types of FAI are cam and pincer. Pincer-type FAI results from increased acetabular depth or overcoverage, while cam-type FAI is a consequence of decreased femoral head–neck offset. The most common location of the cam deformity (asphericity) is at the anterolateral femoral head–neck junction, which increases shear at the chondrolabral junction of the anterosuperior acetabulum during deep flexion and rotational maneuvers. The magnitude of a cam deformity may be measured by a number of imaging parameters. Initially described by Notzli et al. (9) on axial oblique magnetic resonance imaging (MRI) parallel to the plane of the femoral neck, the alpha (α) angle describes where the head–neck junction loses sphericity. The alpha angle has been extrapolated to plain radiographs and computed tomography (CT). In a healthy population, the average α angle is estimated at 42°(9); larger α angles may indicate the presence of a cam.

Cam and pincer morphologies are thought to predominate in men and women, respectively (10–14). The physiologic development of the hip joint differs between males and females, and there are different hypotheses to explain the association (15). Females have earlier closure of the pelvic and proximal femoral physes vs. males (16). In males, the formation and size progression of the cam morphology is around the time of rapid longitudinal growth (ages 12–16) and is associated with impact sports (e.g., hockey, football, basketball, and soccer) (17–19).

The prevalence and characterization of cam morphology is increasingly recognized in males. However, it is underrepresented and potentially unrecognized in females. The purpose of this study is to determine the prevalence of cam morphology in non-arthritic females with symptomatic intra-articular hip pain. The study hypothesis is that the prevalence of female cam impingement is higher than typically reported in the orthopedic literature.

## Materials and Methods

New female patients presenting to the senior author’s office with a chief complaint of “hip pain” between December 2006 and January 2013 were considered. Inclusion criteria included age under 65 years, Tönnis arthritis grade (20) of 0 or 1, adequate anteroposterior (AP) pelvis and lateral (Dunn 90°, cross-table lateral, and/or frog-leg lateral) hip radiographs, and a clinical history and exam consistent with intra-articular hip pathology. Adequacy of AP radiographs was determined by symmetry of obturator foramina, and distance of pubic symphysis and coccygeal tip (separated by 1.5–2 cm). Subjective clinical evaluation consistent with intra-articular pathology demonstrated deep groin pain, worse with deep flexion and rotational maneuvers, worse with sitting rather than standing, pain with putting on socks and shoes, and worse with activity and better with rest. Objective physical examination demonstrated positive impingement testing and decreased hip flexion and internal rotation. Subjects with hip dysplasia (lateral center edge angle <20°, anterior center edge angle <20°, Tönnis angle >10°, or femoral head extrusion index >25%) or prior hip surgery were excluded.

**Figure 1:**
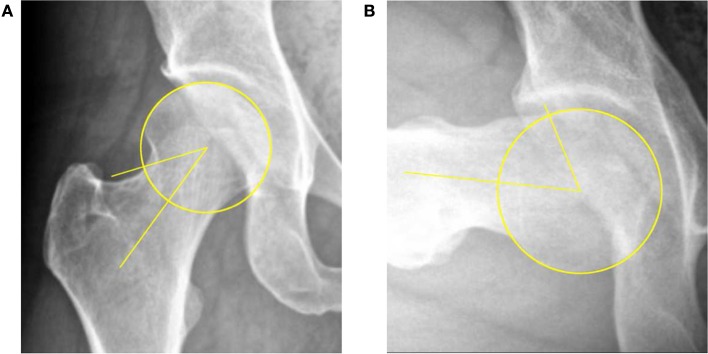
**Determination of head–neck offset by measurement of the α angle on AP (A) and 90°-Dunn lateral (B) radiographs**.

Radiographs were reviewed retrospectively. Tönnis grades were documented and α angles measured on all AP-pelvis and lateral radiographs as described by Notzli et al. (Figure 1) (9). The center of the femoral head, the central axis of the femoral neck, and the resultant α angle were determined using measurement tools available in the MedVIEW Picture Archive Communication System (PACS) software (Aspyra, West Lake Village, CA, USA). Lateral views included frog-leg lateral, cross-table lateral, and/or 90°-Dunn lateral positioning. The largest α angle was used. For each subject, demographic data, including age, ethnicity, and body mass index (BMI), was collected. In order to evaluate the prevalence of cam-type deformity, all patients were classified according to the criteria defined by Gosvig et al. (pathologic > 57° and borderline 51–56°) (21). Additionally, patients were classified as having subtle (46–50°) or very subtle (43–45°) cam morphologies. Normal α angles were defined as ≤42°(9).

Pearson’s correlation was used between α-angle measurements, age, and BMI. Student’s *t*-test was performed to compare α-angle and ethnicity and to compare measurements between different radiographic views. Measurements were performed by two senior resident physicians. An intraclass correlation coefficient (ICC) was found between the two sets of measurements. *p*-Values of <0.05 were considered significant. All statistical tests were performed using SPSS software for Windows, version 13.0 (SPSS, Chicago, IL, USA).

## Results

A total of 969 females were presented to the senior author’s clinic between December 2006 and January 2013 with a chief complaint of “hip pain.” Three hundred and ninety-one patients (438 hips) were eventually diagnosed with FAI and had adequate radiographs for inclusion. The mean age was 36.2 ± 12.3 years (range, 12–66). The mean height was 65.3 ± 8.7 in, weight 145.9 ± 37.1 lbs, and BMI 24.0 ± 5.0 kg/m^2^. Ninety-eight percent were Caucasian. One hundred and ninety-three patients (49.4%) had isolated impingement of the right hip, 151 (38.6%) had isolated left hip impingement, and 47 (12.0%) had bilateral impingement.

Table 1 lists the distribution of the mean largest α angle measured in the study population. The overall mean α angle was 48.2 ± 11.9°. A deformity that was subtle or greater was present in 44.3% of hips, and 64.4% had a deformity very subtle or greater. There was no correlation (*R* = 0.17) between patients’ age and size of the cam lesion nor was there a correlation (*R* = 0.05) between patients’ BMI and size of the cam lesion. There was no difference between the ethnicity of the patient and size of the cam lesion (*p* = 0.10). Interobserver correlation coefficient for α-angle measurements was 0.84.

**Table 1:** **Distribution of α-angle**.

Classification (α-angle)	Number (%)
Pathologic (>57°)	64 (14.6)
Borderline (51–56°)	66 (15.1)
Subtle (46–50°)	64 (14.6)
Very subtle (43–45°)	88 (20.1)
Normal (≤42°)	156 (35.6)

Table 2 lists the distribution of mean α angles measured on the respective views. The mean α angle measured on frog-leg lateral views was significantly greater than that measured on AP, cross-table lateral, and 90°-Dunn lateral views (*p* < 0.001, *p* = 0.02, and *p* < 0.001, respectively). The mean angle measured on AP view was less than that of the cross-table lateral (*p* = 0.09) and significantly less than that of the 90°-Dunn lateral view (*p* < 0.001). There was no significant difference between the cross-table lateral and 90°-Dunn lateral views (*p* = 0.36).

**Table 2:** **Variation of α-angle measurements by radiographic view**.

Radiographic view	Mean α angle (°)	Number of hips in which view showed the largest α angle (%)^a^
Anteroposterior (AP)	41.3 ± 11.0	136 (31.1)
Frog-leg lateral	48.1 ± 12.2	114 (26.0)
Cross-table lateral	43.7 ± 10.3	14 (3.2)
90°-Dunn lateral	44.2 ± 8.6	172 (39.3)

*^a^Two patients had identical α angle measurements on AP and frog-leg lateral views*.

## Discussion

Cam deformities have traditionally been associated with young male athletes, while pincer impingement has been described as a disease of middle-aged women (10–14). The current investigation’s data suggest that there is a significantly higher prevalence of cam deformities found in symptomatic female patients. In this retrospective cohort of 391 women (438 hips) with symptomatic FAI, 29.7% had an α angle >50.5°, compared to just 5.4% of asymptomatic females presented by Hack et al. (12).

The notion that cam lesions occur predominantly in young males is supported by recent literature (12–14, 22). In 2010, Hack et al. evaluated hip MRI in 200 asymptomatic volunteers and 14% of their subjects had cam deformities >50.5°, 79% of whom were male. They reported decreased head–neck offset in just 5.4% of the females enrolled (12). More recently in 2013, Leunig et al. assessed MRIs in 80 asymptomatic females and found 0 cam deformities >57°(13). While these studies suggest that cam deformities are rare in women, they are cross-sectional evaluations of asymptomatic patients and do not represent females who present with symptomatic impingement. Cam lesions can produce significant hip pain and motion restrictions (23, 24), and Miguel et al. have shown that symptomatic patients have significantly higher α angles compared to asymptomatic controls (25). Therefore, the prevalence of cam deformities in asymptomatic females may underestimate the prevalence of such deformities in those with symptoms.

In a recent assessment of FAI morphology in 100 men and women, Nepple et al. found an even greater percentage of cam deformities amongst females symptomatic enough to require surgery (26). Whereas the current cohort included some non-surgical patients successfully treated with physical therapy, Nepple et al. reported that 88% of female patients requiring surgery had an α angle >50°. Of note, they found that, while the majority of both men and women had cam impingement, the mean α angle was greater in men (70.8° vs. 57.6°, *p* < 0.001). Beaule et al. also reported smaller cam lesions in symptomatic females compared to males (*n* = 30, 73.3° vs. 58.7°, *p* = 0.009) (27).

The current study’s findings indicate that symptomatic cam FAI may not be restricted to young males. Moreover, we feel that cam impingement should be thoroughly evaluated in all symptomatic females given the consequences of a missed cam deformity, including continued pain and the possibility of additional surgeries. The leading cause of revision FAI surgery is an inadequate cam resection (28, 29). It is, thus, important to scrutinize the head–neck region in an unbiased fashion and consider a femoral osteochondroplasty for both symptomatic men and women even though it is a technically demanding and time-consuming procedure. With adequate cam resection, both arthroscopic and open hip surgeries have shown excellent short- and midterm outcomes for relieving pain and improving function (30–40).

The α-angle cut-off of 42° for normal female morphology is based on the classification by Gosvig et al. and Notzli et al. (9, 21). This is a conservative threshold compared to the non-gender-specific threshold of 50.5° used in other studies (9, 41–43). The clinical relevance of subtle (46–50°) and very subtle (43–45°) lesions has not yet been established. Abnormal α-angle thresholds in females may need to be lowered compared to male patients to reflect gender-specific pathomechanisms, such as mixed impingement patterns, range of motion differences, and differences in hip girdle musculature (44, 45). Further studies are required to assess the extent of intra-articular pathology associated with these types of lesions and how they may correlate with the risk of developing osteoarthritis.

This study also highlights significant differences in the α-angle measurements depending on the radiographic view. The frog-leg lateral view detected significantly larger cam deformities than each of the other three radiographic views. Clohisy et al. conducted a level II diagnostic study showing that the frog-leg lateral view provides accurate visualization of the femoral head–neck offset when distinguishing symptomatic FAI patients from asymptomatic controls (46). Barton et al. (47) validated both the 90°-Dunn and cross-table lateral views by comparing them to radial oblique reformatted MRI, which has been established as the gold-standard for detecting cam lesions (9, 48, 49). A single AP view is less sensitive at finding cam deformities, which are typically anterosuperior between the 1:30 and 3:00 positions (47–49). The common consensus is that multiple views should be combined to assess multiple planes. In our study, cam lesions were most commonly detected on the most sensitive 90°-Dunn lateral view. The largest respective α angle was found on AP view in 31.1% of hips, but the head–neck offset from these hips was usually classified as normal. It should be noted that these comparisons represent pooled measurements and cannot speak to the accuracy of each radiograph per individual patient; some patients had all four views while others had only two.

To our knowledge, this study represents the largest cohort of symptomatic females evaluated for cam impingement. Radiographs were assessed using a validated system as demonstrated by our high interobserver correlation. Our findings are based on the largest α angles measured from all available radiographs, which minimizes the risk of having missed subtle deformities in different planes. If MRI were available for review, we would have had a greater sensitivity for detecting cam lesions and the prevalence of abnormal α angles may have been even higher than reported.

The limitations of this study are related to its retrospective and cross-sectional design. Therefore, no firm causal inferences can be made. Prospectively collected data from long-term follow-up of cohorts with both genders could clarify the clinical relevance of our findings and whether different degrees of cam deformities are associated with an increased risk of symptomatic hip arthritis. This study also lacks a formal evaluation for pincer lesions, so we cannot make an assessment of the prevalence and clinical relevance of mixed FAI presentations.

## Conclusion

In conclusion, we have found that female patients with symptomatic FAI have a higher prevalence of cam lesions compared to prior reports of asymptomatic females. This may require lower gender-specific radiographic α-angle thresholds to diagnose cam deformities in females. Future studies are required to assess this prospectively and help establish the clinical relevance of these findings.

## Author Contributions

DL: lead author on the study, contributed to all stages of study development. MH: second author, contributed to data analysis and manuscript drafting and editing. JH: third author, contributed to idea of study as well as preliminary data generation and editing of the manuscript. BH: fourth author, contributed to data acquisition ad drafting of manuscript. RF: fifth author, contributed to data analysis and editing of manuscript. SN: sixth author, contributed to study generation and drafting and editing of manuscript.

## Conflict of Interest Statement

The authors did not receive any outside funding or grants in support of their research for or preparation of this work. Neither they nor a member of their immediate families received payments or other benefits or a commitment or agreement to provide such benefits from a commercial entity.
